# Urbanization and mortality in Britain, c. 1800–50†

**DOI:** 10.1111/ehr.12964

**Published:** 2020-02-21

**Authors:** Romola J. Davenport

**Affiliations:** ^1^ Cambridge Group for the History of Population and Social Structure, Department of Geography University of Cambridge

## Abstract

In the long‐running debate over standards of living during the industrial revolution, pessimists have identified deteriorating health conditions in towns as undermining the positive effects of rising real incomes on the ‘biological standard of living’. This article reviews long‐run historical relationships between urbanization and epidemiological trends in England, and then addresses the specific question: did mortality rise especially in rapidly growing industrial and manufacturing towns in the period *c*. 1830–50? Using comparative data for British, European, and American cities and selected rural populations, this study finds good evidence for widespread increases in mortality in the second quarter of the nineteenth century. However, this phenomenon was not confined to ‘new’ or industrial towns. Instead, mortality rose in the 1830s especially among young children (aged one to four years) in a wide range of populations and environments. This pattern of heightened mortality extended between *c*. 1830 and *c*. 1870, and coincided with a well‐established rise and decline in scarlet fever virulence and mortality. The evidence presented here therefore supports claims that mortality worsened for young children in the middle decades of the nineteenth century, but also indicates that this phenomenon was more geographically ubiquitous, less severe, and less chronologically concentrated than previously argued.

Until the twentieth century, death rates were generally higher in urban areas compared with rural ones, a phenomenon dubbed the ‘urban penalty’. Urban death rates were high partly as a consequence of factors that can be considered as structural features of cities and towns.1
Landers, ‘Historical epidemiology’. High population densities favoured the transmission of infectious diseases, and trade and migration promoted the importation of animal and human diseases. In addition, before the twentieth century most cities provided inadequate facilities for the disposal of the volumes of wastes generated by such densities and numbers of humans and animals, and for the prevention and treatment of gastrointestinal diseases associated with these living conditions. However, while these factors were probably ubiquitous among historical towns and cities, there were large variations in mortality rates, both chronologically and geographically, that reflected more contingent influences. These included wider epidemiological factors such as the particular array of diseases present, and autonomous variations in pathogen characteristics. The extent to which rural migrants to cities were vulnerable to urban diseases also varied depending on their exposure to the same diseases prior to migration, and therefore on the degree to which rural and urban populations were integrated into common disease pools.2
McNeill, ‘Migration’; Davenport, Boulton, and Schwarz, ‘Adult smallpox’. Distinctive urban cultural practices could also exert profound influences on mortality; for example, where infants were wet‐nursed or hand‐fed in preference to maternal breastfeeding.3
Landers, *Death*, p. 152; Sussman, ‘Parisian infants’.


As a consequence of these various factors, urban populations almost everywhere experienced higher rates of communicable diseases than their rural hinterlands before the twentieth century. However, the extent of the gap varied considerably over time. For much of the seventeenth and eighteenth centuries, cities in Europe appear to have functioned as demographic sinks, reliant on immigration to balance very high death rates.4For discussions, see de Vries, *European urbanisation*, ch. 9; Galley, ‘Model’; van der Woude, ‘Population developments’. However, by *c*. 1800 cities in Britain and parts of north‐western Europe were largely capable of sustaining and increasing their population sizes through natural growth.5For example, Mercer, *Disease*, ch. 4; Sharlin, ‘Natural decrease’; van der Woude, ‘Population developments’. The rural–urban gap diminished rapidly in the late nineteenth and early twentieth centuries, and in Britain urban life expectancies converged with rural ones in the 1930s and then overtook them, a phenomenon that is now global.

This article first reviews long‐run historical relationships between urbanization and epidemiological trends in England (section I), and then focuses on the period *c*. 1830–50, when mortality apparently rose again in towns. It provides new evidence for such a reversal (sections II–IV), and, critically, demonstrates that it occurred in rural as well as urban populations in Britain and elsewhere, and was sustained until *c*. 1870. These patterns are difficult to explain as a function of industrialization and worsening urban conditions. Instead, attention is drawn to the marked similarities in the age pattern and timing of this heightened mortality, and the rise and fall of mortality caused by apparently autonomous changes in the virulence of the causal agent of scarlet fever (section V). Section VI concludes.

## I

**Figure 1:**
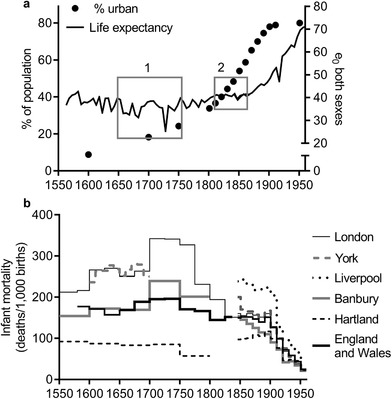
Percentage of the population of England and Wales living in settlements of population 2,500 or more, and life expectancy at birth, England and Wales, sexes combined (panel a), and infant mortality (deaths in the first year of life per 1,000 births) (panel b) *Sources*: *8th to 23rd Annual Reports of the Registrar‐General* (P.P. 1847/8, XXV; 1849, XXI; 1850, XX; 1851, XXII; 1852, XVIII; 1852/3, XL; 1854, XIX; 1854/5, XV; 1856, XVIII; 1857, XXII; 1857/8, XXIII; 1859, XII; 1860, XXIX; 1861, XVIII; 1862, XVII); Bennett, ‘Urban population database’; de Vries, *European urbanization*, p. 64; Galley, *Demography*, tab. 4.9; *Human mortality database*; Landers, *Death*, p. 136; Newton, ‘Infant mortality’; Smith, ‘Population’, p. 210; University of Portsmouth, ‘A vision of Britain through time’ [website], http://www.visionofbritain.org.uk/ (accessed on 1 Oct. 2011); Woods, ‘Causes of death’; Wrigley, Davies, Oeppen, and Schofield, *English population history*, tab. A9.1; Wrigley and Schofield, ‘Population history’.

We can examine the long‐run impact of urbanization on national life expectancy in the case of England, the only country for which we have robust national estimates of life expectancy over the last 500 years. In this period England was transformed from a very lightly urbanized country, relative to the European average, into the world's first truly urban nation. De Vries estimated that in 1600, 7.7 per cent of the population of England and Wales lived in towns with a population of 5,000 or more, compared with 10.8 per cent for western Europe as a whole.6
de Vries, *European urbanisation*, pp. 65, 76. Urbanization proceeded across the period 1600–1800, but accelerated in the first half of the nineteenth century (figure 1a). By 1851 over half the population lived in settlements of 2,500 or more, peaking at around 80 per cent by the 1890s.

Figure 1a also plots long‐run life expectancy in England and Wales. A comparison between the plots suggests that there was no consistent relationship between urbanization and life expectancy. Despite the progressive urbanization of the English population over the last half millennium, average life expectancy appears to have worsened in only one period, *c*. 1650–1750 (box 1 in figure 1a).7Wrigley and Schofield estimated that life expectancy fell 7–8 years between the late sixteenth and the late seventeenth centuries, and did not improve decisively until the mid‐eighteenth century; Wrigley et al., *English population history*, p. 348. The rapid urbanization of the early nineteenth century was apparently accompanied by a stabilization of mortality (box 2) with little net change until the onset of secular mortality decline from the 1870s. Figure 1a implies therefore that the movement of population into towns was accompanied in most periods by improvements in average life expectancy sufficient to balance, and in some periods to outweigh, the higher mortality of urban areas.

Between the mid‐seventeenth and the mid‐eighteenth century in England life expectancy fell to its lowest point in the entire period between 1540 and the present (box 1, figure 1a). This fall occurred despite comparatively high real wages and negligible population growth. Wrigley and Schofield attributed this decidedly non‐Malthusian deterioration in life expectancy to increases in disease exposure associated with economic integration and urbanization.8
Wrigley and Schofield, *Population history of England*, pp. 412–17. Alternatively, or in conjunction, smallpox may have made a major new contribution to mortality in this period, as a consequence both of increasing contacts between populations, and possibly of the emergence of a novel strain of the virus.9
Carmichael and Silverstein, ‘Smallpox’; Davenport, Boulton, and Schwarz, ‘Urban inoculation’; Duggan et al., ‘17th century variola virus’. Mortality also rose within towns in this period. Indeed urban death rates mirrored national patterns in exaggerated form. Figure 1b shows infant mortality rates rather than life expectancies, because the latter require much more data and are rarely available for urban populations before the mid‐nineteenth century. However, levels of infant mortality were so high in early modern towns and cities that mortality in the first year of life was a major driver of life expectancy levels, at least in the eighteenth century. In London infant mortality was around 300–400 deaths per 1,000 births in the mid‐eighteenth century, compared with the national average of *c*. 180 per 1,000. While London was then the largest city in Europe, with a population of perhaps *c*. 700,000, even small market towns seem to have experienced a severe ‘urban penalty’ in this period. In the towns of Alcester, Banbury, Gainsborough, and Lowestoft, with populations of 2,000–3,000, infant mortality was in the range 209–270 per 1,000 in the period 1675–1749, compared with infant mortality rates below 100 per 1,000 in the most remote rural parishes.10
Wrigley et al., *English population history*, pp. 270–1.


In contrast to the period 1650–1750, the early stages of the classic industrial revolution period, *c*. 1760–1820, were associated with *rising* life expectancy at the national level, despite rapid urbanization. This improvement in survival was most marked in urban populations, as indicated by infant mortality rates (figure 1b). The result was a remarkable geographical convergence in infant mortality between the mid‐eighteenth and the mid‐nineteenth century.11
Galley and Shelton, ‘Bridging the gap’. By 1850, when demographic data for towns are more abundant, even the most notorious Victorian cities, Liverpool and Manchester, reported infant mortality rates no higher than those of small market towns a century earlier.

The causes of this dramatic improvement in urban life expectancy after *c*. 1750 remain unclear. The progressive control of smallpox by isolation and inoculation in the eighteenth century, and the extraordinary success of smallpox vaccination after *c*. 1800, played a significant role.12
Davenport et al., ‘Urban inoculation’; Davenport, Satchell, and Shaw‐Taylor, ‘Geography’; Mercer, *Disease*, ch. 3. However, other factors, including changes in infant feeding practices, also appear to have been important in London and probably other urban centres.13
Davenport, ‘Infant feeding practices’; Landers, *Death*, p. 153.


The profound improvements in life expectancy in cities and towns in the late eighteenth and early nineteenth centuries remain rather poorly appreciated. Indeed it is sometimes assumed that urban mortality reached an apogee in the nineteenth century as a consequence of industrialization and unconstrained urban growth.14For example, Cutler and Miller, ‘Public health’; Oris and Fariñas, ‘New approaches’, pp. 5–6; Szreter, ‘Population health’, p. 424. This perception is profoundly at odds with the history of urban demography. In western Europe the nineteenth century was a period when cities moved decisively from being demographic sinks dependent on immigration to sustain their population size, to being self‐reproducing populations capable of natural growth. For example, even Stockholm, perhaps the unhealthiest city in northern Europe in the nineteenth century, enjoyed significantly higher life expectancies in the nineteenth century than the eighteenth.15
Woods, ‘Historical relationship’. See also fig. 7. While it remains unclear whether the ‘urban graveyard’ model applied to all cities before the nineteenth century or was predominantly an early modern phenomenon, it is obvious that the industrial cities of the nineteenth century would have constituted a major drain on the national population had they grown to prominence a century earlier.16
de Vries, ‘Problems’; Wrigley, ‘Simple model’.


A second aspect of urban mortality that is often overlooked is that despite exhibiting the highest rates of urbanization and industrialization in the world, death rates in English cities in the mid‐nineteenth century were modest by the standards of many continental European cities. This was particularly the case with respect to infant mortality, but mortality at older ages was also unexceptional. London, then the largest city in Europe, was extraordinarily salubrious compared with much smaller British and continental cities.17
Luckin and Mooney, ‘Urban history’. However, even the unhealthiest British cities, including Liverpool, Manchester, and Glasgow, were unremarkable by continental standards.18For example, Floris and Staub, ‘Swiss towns’; Fridlizius, ‘Malmö’; Kearns, Lee, and Rogers, ‘Interaction’; Peltola and Saaritsa, ‘Finnish cities’; Preston and van der Walle, ‘French mortality’; Söderberg, Jonsson, and Persson, *Stagnating metropolis*, pp. 176–7; Vögele, *Urban mortality*, ch. 5.


After 1820 life expectancy in England and Wales as a whole apparently stagnated until the 1870s, when it embarked on a secular rise that has continued to the present (figure 1a). This period of stasis in the nineteenth century has been attributed to the braking effects of urbanization on an underlying process of continuing mortality decline.19See Torres, Canudas‐Romo, and Oeppen, ‘Contribution of urbanization’, for an elegant demonstration of the impact of urbanization on Scottish life expectancies, 1861–1910. The exact trajectory of national life expectancy in the first half of the nineteenth century is, however, unclear. Wrigley and Schofield's life expectancy estimates were generated using generalized inverse projection, which used mortality schedules from family reconstitution studies for the period before the inception of civil registration in 1837.20Wrigley et al., *English population history*, pp. 613–16. These studies included rural and small urban populations but omitted large towns, which are too difficult to reconstitute in this period. London was included in the projection estimates, but it remains likely that the reconstitution sample underestimated national mortality rates, especially for young children, the age group most affected by urban conditions.

**Figure 2:**
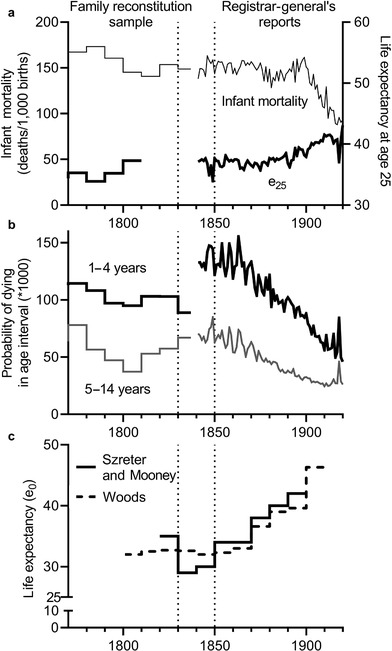
Long‐run trends in infant mortality and life expectancy at age 25 (e_25_) (panel a), and child mortality (panel b), and estimates of life expectancy at birth in large towns (panel c) *Note*: Vertical dashed lines indicate the period under debate. *Sources*: Wrigley et al., *English population history*, p. 93 (pre‐1838 data in panels a and b); *Human mortality database* (post‐1840 data in panels a and b); Woods, *Demography*, tab. 9.4, p. 369 (panel c).

Figures 2a and b compare national mortality rates derived by Wrigley and Schofield from parish registers, and the Registrar‐General's measures from 1841 onwards. In the case of infant mortality rates the fit between the two series is very good (figure 2a). Mortality rates for older children (aged five to 14 years) and expectation of life for young adults (at age 25) also dove‐tailed very neatly with series derived from the Registrar‐General's data (figure 2a and b). However, for young children (in the age range of one to four years), the Registrar‐General's rates were significantly higher than the rates recorded in the Cambridge Group parish sample (figure 2b).21See also Woods, *Demography*, pp. 251–5; Wrigley et al., *English population history*, pp. 258–61. This is the age group most affected by urban living conditions, and for which mortality was most clearly related to population density.22
Woods and Shelton, *Atlas*, pp. 56, 59; Woods, *Demography*, p. 196. See also fig. 4.


Wrigley and others have, however, concurred that the more likely explanation for this apparent discontinuity is that early childhood mortality rates worsened in the period after 1820.23
Woods, *Demography*, p. 254; Wrigley et al., *English population history*, pp. 258–61. Wrigley and colleagues drew attention to a simultaneous rise in early childhood mortality in Sweden, and evidence for (smaller) rises in infant mortality in London, Glasgow, and nine industrializing parishes in northern England, and a rise in mortality at ages under five years in Carlisle.24Wrigley et al., *English population history*, pp. 255–61. In the reconstitution sample there was also a marked rise in mortality among older children (aged five to 14 years) in the closing decades of the reconstitution period (1810–37), although the increase in this age range never exceeded mid‐eighteenth‐century levels, and was concordant with levels in the national population reported by the Registrar‐General after 1837 (figure 2b). Wrigley et al. suggested in passing that the apparent upturn in childhood mortality in the middle decades of the nineteenth century reflected ‘changes in the type and virulence of prevalent fatal diseases’.25Ibid., p. 260. This raises the question of whether existing life expectancy estimates for this period are too high, because they are based on a melding of parish reconstitution and national data. However Wrigley et al. took into account their evidence of rising child mortality in calculating life expectancy estimates for the early and mid‐nineteenth century, on the assumption that the reconstitution sample reflected the trend if not the magnitude of worsening, and applied this trend to their estimates based on the 3rd English life table; ibid., pp. 522–3. Other proponents of the view that infant and child mortality worsened abruptly in the early nineteenth century emphasized the particular lethality of towns for young children in this period; however, they disagreed on the causes of these patterns.26
Armstrong, ‘Trend’; Huck, ‘Infant mortality’; Kitson, ‘Industrialization’; Levine, *Family formation*, pp. 68–73; Szreter and Mooney, ‘Urbanisation’.


The question of whether mortality worsened at the national level over the period *c*. 1820–50 remains unresolved because we have almost no evidence regarding the trajectories of life expectancy in large towns. Indeed the declining reliability of Anglican registers, the proliferation of non‐conformist sects, and the rarity of cause of death data following the standardization of burial registration in 1813 led Galley to dub the period *c*. 1750–1850 ‘a dark age of urban demography’.27Quoted in Sharpe, ‘Population’, p. 491. This is particularly unfortunate because this was a key period of urbanization and industrialization, and one characterized by apparently large changes in mortality patterns.

In the light of this paucity of data, two major interpretations of urban mortality in this period have been proposed. In the first case, Szreter and Mooney claimed that the period from *c*. 1830 to the mid‐1850s marked a particular nadir in urban mortality, with life expectancy in large towns (with populations above 100,000) averaging 29–30 years in the 1830s and 1840s (figure 2c).28
Szreter and Mooney, ‘Urbanisation’. Note that even these dire life expectancies compared favourably with Landers’s (*Death*, pp. 158, 171) estimates of 18–22 years for London in the early and mid‐eighteenth century. Szreter and Mooney derived their life expectancy estimates for large towns in the 1830s solely from estimates based on the bills of mortality for Glasgow, and for the 1840s from two life tables for Liverpool and Manchester, published in the early reports of the Registrar‐General. The alleged fall in life expectancy in large towns between the 1820s and the 1830s therefore relied solely on such a trend in the Glasgow bills of mortality. Szreter and Mooney illustrated their argument with examples of worsening mortality in the northern town of Carlisle and in a sample of industrializing villages in northern England. This phenomenon of falling life expectancies was, they argued, a feature of rapidly growing northern towns, and was confined largely to the second quarter of the nineteenth century. While they suggested that conditions ameliorated only from the late 1850s,29
Szreter and Mooney, ‘Urbanisation’, p. 98. in fact their very low estimates of life expectancy in the largest towns related to the decades of least data availability, 1830–49 (figure 2c; the dashed lines in all three panels indicate the decades of the 1830s and 1840s).

Acknowledging that urban growth and industrialization were extremely rapid in the period 1780–1820 without any worsening of urban mortality, Szreter argued that the political conditions of the period *c*. 1820–50, and in particular the uncoupling of the common political interests of small employers and workers, resulted in administrative failures in rapidly growing towns that lacked a long‐established administrative infrastructure.30
Szreter, ‘Economic growth’. The deterioration in mortality after 1820 was, according to Szreter, driven in the main by a rise in mortality from ‘infectious diseases and sanitation diseases, especially at ages 3–24 months’.31Ibid., p. 700. He attributed this rise to ‘ratepayer “economy”’ and laissez‐faire attitudes to infrastructure provision, vaccination and housing that had their greatest impact in fast‐growing industrial towns.32Ibid., p. 710. Szreter went so far as to generalize from the British experience to argue that rapid economic growth inevitably produced disruption and deprivation, with potentially negative consequences for health.33Ibid., p. 715; idem, ‘Population health’.


In contrast to the views of Szreter and Mooney, Woods proposed a more benign interpretation of demographic developments in the early nineteenth century. He suggested that there was a much more modest rise in urban mortality rates in the second quarter of the nineteenth century (figure 2c), and he argued that this was not a function of local political factors but was the inevitable epidemiological consequence of urbanization.34
Woods, *Demography*, pp. 368–71. See idem, ‘Population redistribution’, for his earlier view that mortality was fairly stable throughout the period *c*. 1800–70. The impact of urbanization took two main forms. First, before the disappearance of the urban penalty, urbanization necessarily entailed the redistribution of population from relatively low‐mortality rural areas to higher‐mortality urban environments. This compositional shift should have raised mortality at the national level, without any necessary change in local mortality rates.35
Woods, *Demography*, pp. 368–71. However, Woods also argued that some additional increase in mortality would be expected as towns grew, because some diseases (including measles and most ‘childhood’ diseases) increased in impact with increasing population size. In these cases, larger populations supported more frequent epidemics, raising the average level of exposure to the disease and lowering the average age at infection. Therefore urbanization would be expected to be accompanied by rising mortality, regardless of living conditions or nutrition, unless there were strong countervailing factors to drive mortality down. Such countervailing factors were clearly present in the period 1750–1820, but these appear to have lost their force after *c*. 1820.

Woods also invoked autonomous factors, in particular a documented rise in the virulence of scarlet fever as a cause of rising mortality especially among young children. A second arguably autonomous factor was the importation of cholera in 1831–2, 1848–9, 1854, and 1866. Cholera caused very large epidemics in these years, although the strictly demographic impact was small. Overall, Woods did not accept that local policies were the cause of any increases in mortality in the first half of the nineteenth century, and suggested that excess mortality in certain northern towns was a function of the poverty of Irish immigrants.36Ibid., p. 370, n. 16.


Here we take three new approaches to improve our understanding of urban mortality patterns in England in the first half of the nineteenth century. First, we use the only series we have for England that effectively span this period, the reconstitution parishes and their associated registration districts used by Wrigley et al. (section II). We find that mortality rose at ages one to four years in the 1830s in most settlement types in our sample, suggesting that this phenomenon was not restricted to large towns, or to fast‐growing industrial and manufacturing towns. We have no comparable data before 1838 for large towns, and therefore the second approach (section III) utilizes the Registrar‐General's early reports of mortality in registration districts (1838–44) to derive life expectancy estimates for the largest towns (excluding London) and to create time series of infant and early childhood mortality rates (1838–1910). These series provide little evidence that mortality was substantially worse in the late 1830s and 1840s compared with the 1850s or 1860s. To test the possibility that urban mortality rates did indeed worsen between the 1820s and the 1830s, when we have no data for English cities, the third approach compares nineteenth‐century mortality trends in rural and urban populations outside Britain (section IV). Here we find substantial evidence for increases in early childhood mortality in the period *c*. 1830–50 in both rural and urban populations. Section V assesses scarlet fever as a contributory factor to these trends, and section VI concludes. Taken together our findings refute the specific claims made by Szreter and Mooney, that mortality worsened particularly in fast‐growing industrial and manufacturing towns and was most extreme in the decades of the 1830s and 1840s. Instead they support, and extend, Woods's interpretation of both the trends and the causes of urban mortality patterns in this period.

This article omits discussion of two major issues regarding mortality patterns in British towns in this period: sanitary conditions and public health measures. Sanitary conditions and faecal‐oral disease rates are addressed in a companion paper.37Davenport, Satchell, and Shaw‐Taylor, ‘Cholera’. Urban public health policy is a very large and complex issue, and even where it is possible to quantify effort in this period (for example, through expenditure or infrastructure) then the connections between interventions and mortality outcomes may be tenuous or difficult to detect.38
Ibid.; Alsan and Goldin, ‘Watersheds’; Harris and Hinde, ‘Sanitary investment’; van Poppel and van der Heijden, ‘Water supply’. See also Chapman, ‘Contribution of infrastructure’. This study instead takes a comparative approach to test whether outcomes (mortality rates) responded similarly in this period across a range of towns and other settlement types with very different socioeconomic characteristics and public health policies.

## II

This section combines three sources of information on mortality rates to assess whether mortality rose in the period 1830–50. These are the parish family reconstitution data created by Wrigley et al.; the very earliest mortality rates reported by the Registrar‐General, from 1838–50; and decadal mortality rates for the years 1851–1910.39
*8th to 23rd Annual Reports of the Registrar‐General* (P.P. 1847/8, XXV; 1849, XXI; 1850, XX; 1851, XXII; 1852, XVIII; 1852/3, XL; 1854, XIX; 1854/5, XV; 1856, XVIII; 1857, XXII; 1857/8, XXIII; 1859, XII; 1860, XXIX; 1861, XVIII; 1862, XVII); Woods, ‘Causes of death’; Wrigley et al., *English population history*, p. 93.


**Figure 3:**
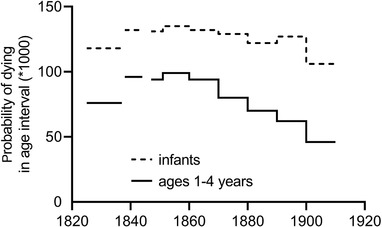
Infant and early childhood mortality in reconstitution sample parishes and associated registration districts (unweighted means), 1825–1910 *Sources*: See tab. 1.

As discussed earlier, Wrigley et al. argued that the discrepancy between their estimates of early childhood mortality for the decades before 1837 and the Registrar‐General's for the period after 1837 (figure 2b) reflected an abrupt rise in mortality in this age group, rather than a deficiency of their sample. Wrigley et al. based their estimates of trends in early childhood mortality on family reconstitutions of parish register data. Their sample consisted, for the early nineteenth century, of eight parishes, and included two small market towns, two industrializing villages, and four parishes of mixed or rural economic activities.40
Wrigley et al., *English population history*, pp. 44–51. Wrigley et al. published infant and early childhood mortality rates in the individual parishes in their sample for the end of the parish register period, 1825–37. They compared these with the earliest age‐specific mortality rates published by the Registrar‐General, for the period 1838–44.41Ibid., p. 93. The smallest spatial units for which the Registrar‐General reported mortality in this period were not parishes but registration districts, which were much larger units comprised of multiple parishes. Therefore the comparison of mortality rates presented in table 1 relies on comparison of parishes with the much larger units of registration districts in which the reconstituted parishes sat. The unweighted mean rates for the eight parishes and associated units are displayed in figure 3.

**Table 1:** Infant and early childhood mortality in reconstitution sample parishes and associated registration districts, 1825–1900

Parish 1825–37	Ash	Banbury	Bottesford	Dawlish	Gedling	Morchard Bishop	Odiham	Shepshed
Parish type	Agricultural	Market town	Mixed	Market town	Industrial village	Agricultural	Mixed	Industrial village
RD 1847–1910^a^	Eastry^b^	Banbury^c^	Grantham^d^	Newton Abbot	Basford	Crediton^e^	Hartley Wintney^f^	Loughborough^g^
*Infant mortality rate*								
1825–37	106	148	134	84	140	82	74	179
1838–44	122	152	138	97	161	89	106	189
1847–50	107	134	149	116	176	91	96	180
1851–60	122	142	148	122	180	88	101	180
1861–70	110	127	139	125	165	102	111	174
1871–80	109	125	128	122	168	96	107	176
1881–90	108	115	134	117	157	91	105	149
1891–1900	111	110	134	125	162	95	124	156
Early childhood mortality rate (_4_q_1_ * 1,000)								
1825–37	78	107	57	85	82	48	65	85
1838–44	81	103	90	103	112	86	74	119
1847–50	74	105	81	140	100	81	67	103
1851–60	85	102	88	90	121	82	101	121
1861–70	90	99	82	105	117	73	76	112
1871–80	62	77	81	83	107	58	66	103
1881–90	52	69	69	71	95	65	54	87
1891–1900	54	49	57	65	85	54	58	71
*Population density (persons/km^2^)*								
Parish, 1841	74	530	68	164	145	67	101	181
RD, 1861	131	101	71	134	2,887	53	115	137

*Notes: a* For the years 1838–44 (and 1845–6) the Registrar‐General reported deaths for groups of registration districts, except for the largest towns. From 1847 deaths were reported for individual registration districts (RDs).

*b* Isle of Thanet and Eastrey registration districts in 1838–44.

*c* Woodstock, Bicester, and Banbury in 1838–44.

*d* Grantham and Sleaford in 1838–44.

*e* Crediton, South Molton, Barnstaple, and Torrington in 1838–44.

*f* Hartley Wintney, Alton, Basingstoke, Alresford, and Petersfield in 1838–44.

*g* Loughborough and Barrow‐on‐Soar in 1838–44.

*Sources*: Wrigley et al., *English population history*, p. 93; *8th–11th Annual Reports of the Registrar‐General* (P.P. 1847/8, XXV; 1849, XXI; 1850, XX); Woods, ‘Causes of death’.

The mortality series for individual parishes and their registration districts followed the national pattern fairly closely. Mortality was generally higher in the registration districts in the period 1838–44 compared with parishes in the period 1825–37, and this was more marked among those aged one to four years than in infancy. A number of the registration districts in which the reconstitution parishes were located apparently experienced much higher childhood mortality than the parishes in the preceding decades. This was the case for the isolated rural Devon parish of Morchard Bishop, as well as for the industrializing villages of Shepshed (Leicestershire) and Gedling (Nottinghamshire), the market town of Dawlish (Devon), and the mixed economy parish of Bottesford (Leicestershire). Changes in early childhood mortality after *c*. 1837 were relatively slight in the case of Ash (Kent, agricultural) and Odiham (Hampshire, mixed economy), and early childhood mortality apparently fell slightly in Banbury (Oxfordshire), the largest parish in the reconstitution sample and a market town.

There are three obvious potential explanations for these patterns in early childhood mortality, two of which were discussed earlier with respect to the same feature in the national pattern. First, the differences between the reconstitution sample parishes and their registration districts may be due to the more urban nature of some of the registration districts compared to the parishes; second, there may have been a sudden rise in mortality in this period; or third, the data may be faulty in some way.42An additional potential cause of discrepancy between the two sources of demographic data is that the reconstitution data refer to legitimate infants and children, whereas rates for registration districts include illegitimate infants and children. However, adjustments for higher mortality among illegitimate infants reduced but did not eliminate the discrepancy; Wrigley et al., *English population history*, p. 96. Fig. 2a includes data for illegitimate and legitimate infants. We consider the latter explanation first. Briefly, the early returns to the Registrar‐General of births and deaths have been subject to repeated scrutiny, and are considered to have been deficient with respect to the reporting of both births and especially early neonatal deaths, although the extent of under‐reporting varied geographically.43
Woods, *Demography*, pp. 38–70. These deficiencies are considered to have persisted in diminishing form into the 1860s. Therefore comparisons of infant mortality in particular may be affected by differences in levels and trends in under‐reporting. Liverpool, for example, was considered to have suffered from marked under‐reporting of live births, a tendency that would have acted to inflate infant mortality rates by artificially reducing the denominator for the rate.44Ibid., pp. 31, 60. However, these problems should not have affected the measurement of early childhood rates (ages one to four years), because these do not depend on the scrupulous registration of births or of neonatal deaths. Age reporting in the census was also subject to age heaping and to more serious misreporting especially among young adult women. There was some evidence of misreporting of one‐year‐olds as two‐year‐olds in the census, but this should not have affected the calculation of mortality rates for the one‐to‐four‐years age group as a whole.45Ibid., p. 67. Since this is the age group that demonstrated the most marked discontinuities between the parish sample and the registration district sample in table 1, data quality probably does not account for the observed pattern.

**Figure 4:**
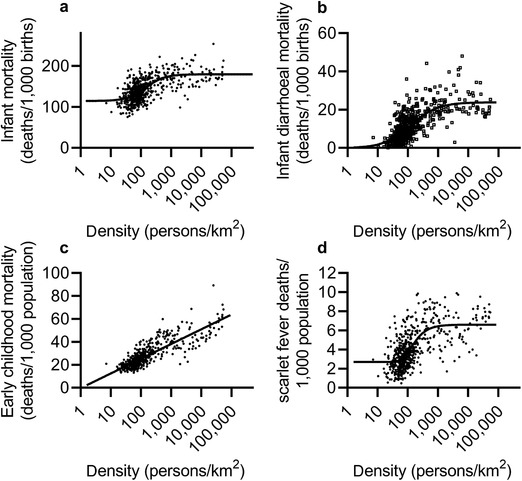
Mortality in registration districts by population density, England and Wales, 1861–70 *Notes*: Rates presented are for infant mortality (panel a), diarrhoeal mortality in infants (panel b), early childhood mortality at ages 1–4 years (panel c), and scarlet fever mortality at ages 1–4 years (panel d). Data were fitted with asymmetric sigmoidal functions (a, b, d) or a linear function (c). *Source*: Woods, ‘Causes of death’.

Was infant and early childhood mortality higher in the period after 1837, in table 1, because the registration districts used to proxy the parishes in this period were more urban in composition than the parishes, and therefore had higher average mortality rates? We tested this possibility by comparing the population densities of the parishes in 1841 with the population densities of the registration districts in 1861. Both infant and childhood mortality rates were sensitive to population density across the second half of the nineteenth century (at least at the level of registration district). However infant mortality, and diarrhoeal mortality (a major cause of excess infant mortality in towns), displayed a sigmoidal relationship to population density, with greater responsiveness to density effects at relatively low densities, and very little further effect of density above a fairly low threshold (figure 4a and b). In contrast, early childhood mortality displayed a more linear relationship to density (figure 4c). Therefore we might expect any increases in population density (between samples or over time) to have had the greatest effect on young children (but not infants), because they shifted the population upward along the mortality curve depicted in figure 4c.46By contrast adult mortality (ages 25–64) was relatively insensitive to population density; ibid., pp. 194–8.


Surprisingly, we found very little difference in population densities between the parishes and their registration districts (last two rows in table 1). The main exception was the large difference in population density between the industrializing village of Gedling (in Nottinghamshire, with a population density of 145 persons per km^2^ in 1841) and its registration district of Basford, which included part of Nottingham town and had an average population density of 2,887 persons per km^2^ in 1861. In the case of the market town of Banbury the population density of the registration district of the same name was significantly lower than the parish as a result of the inclusion of rural parishes around Banbury, a factor that may have contributed to the almost negligible discrepancy in early childhood mortality rates between the two units. Otherwise, however, differences in population densities between parishes and registration districts were very small. Therefore discrepancies in the levels of urbanization in the two samples (before and after 1837) are unlikely to explain the discontinuities in mortality rates between the two periods.

We are therefore left with the explanation offered by Wrigley et al. for the discontinuities in mortality series at the national level *c*. 1837: that there was an abrupt rise in mortality, especially affecting young children, in this period. This is consistent with the findings of Szreter and Mooney. However, this discontinuity was evident in relatively slow‐growing and rural populations in southern England (Dawlish, Morchard Bishop) as well as midlands and industrializing parishes and registration districts in the sample (table 1). This suggests that any epidemiological factors driving a sudden rise in mortality may have been of fairly widespread geographical effect.

The second important point evident from figure 3 is that any increase in mortality that occurred in the 1830s or 1840s was apparently sustained until at least the 1860s in the reconstitution parishes and their associated registration districts. That is, there was no evidence in this sample that mortality was especially elevated in the period for which our evidence base was especially fragile (*c*. 1830–50).

## III

What of mortality trends in towns? For large towns we have no comparable data for the period before 1838. However, we could use the Registrar‐General's returns for the period 1838–1900 to assess the argument that mortality in large towns was excessive in the period between 1838 and *c*. 1850, and then declined. In this section we examine mortality trends in the 14 largest towns in England (excluding the metropolis).47We omit London because its size and heterogeneity meant that it required a separate treatment. See Szreter and Mooney, ‘Urbanisation’, p. 92. These were all towns that were close to or had exceeded the threshold of 100,000 by 1870.48The Registrar‐General included 16 towns in his list of ‘Great Towns’ in 1870 but we omitted Leeds and Portsmouth because the Registrar‐General did not report deaths separately for the ‘core’ districts of these towns (Leeds and Portsea) before 1847 but instead included them with the registration districts of Hunslett and Alverstoke respectively. Since we could not construct comparable aggregates of registration districts for the other 14 towns in the same way (see section III), it was preferable to exclude these two towns. We followed Szreter and Mooney in examining only the ‘core’ registration districts associated with these towns (for example, Liverpool but not West Derby), because the other districts that were associated with the towns by 1870 were reported in aggregate with other districts before 1847, and also because they were more rural than the core districts especially in the 1840s and would have biased mortality rates for the towns downward in this decade relative to later decades.49For example, in 1871 the borough (which became the urban district) of Liverpool included the complete population of Liverpool registration district (238,411), and 74% of the population of West Derby registration district (254,994 out of a total population of 342,925). The relationship of urban districts to their constituent registration districts was first set out clearly in the 1881 census (which also reported retrospective data for 1871); *Census of England and Wales 1881*, vol. II (1883), p. 449. Our measures include life expectancy at birth, and mortality in the first five years of life.

Table 2 displays mortality rates for infants and young children. Rates in italics indicate those towns where mortality declined by at least 10 per cent between 1838–44 and the 1860s. Infant mortality rates showed muted change over the period, and may have been affected (inflated or deflated) by under‐registration of births and infant deaths especially before the 1860s (as discussed in the previous section). However, early childhood mortality rates were less subject to these problems, and revealed a mixed picture. Mortality fell after the 1840s in Bristol, Hull, Leicester, Manchester, Norwich, Nottingham, and Salford. On the other hand, early childhood mortality was fairly stable or rose in the period 1838–70 in Birmingham, Bradford, Liverpool, Newcastle, Sheffield, Sunderland, and Wolverhampton. The towns where early childhood mortality fell between 1845 and 1870 (consistent with the argument that mortality was excessive in the decades of the 1830s and 1840s) included both ‘old’ towns, such as Bristol, Norwich, Nottingham, and Leicester, and exemplars of rapid industrialization, including Manchester and Hull. Early childhood mortality fell in all the large towns after 1870.

**Table 2:** Infant and early childhood mortality in core registration districts of large towns, 1838–1900

Period	1838–44	1845–50	1851–60	1861–70	1871–80	1881–90	1891–1900
*Infant mortality rate*							
Birmingham	187	180	179	177	179	174	200
Bradford	186	205	196	192	177	161	174
Bristol	178	171	171	172	168	156	160
Hull	*265*	*238*	*195*	*181*	178	167	189
Leeds	184	202	189	195	189	174	179
Leicester	198	209	202	211	214	203	195
Liverpool	236	224	241	234	217	219	223
Manchester	*235*	*235*	*216*	*200*	197	193	211
Newcastle	200	205	190	187	176	162	173
Norwich	*249*	*194*	*197*	*182*	188	166	181
Nottingham	*228*	*219*	*213*	*186*	184	174	186
Salford	*206*	*202*	*193*	*174*	184	183	206
Sheffield	185	191	187	192	183	176	195
Sunderland	180	183	164	164	166	160	176
Wolverhampton	*193*	*219*	*197*	*173*	161	161	188
Early childhood mortality (_4_q_1_ * 1,000)							
Birmingham	191	206	200	204	166	137	135
Bradford	157	192	187	169	159	121	116
Bristol	*227*	*199*	*188*	*200*	149	136	108
Hull	*193*	*212*	*164*	*166*	133	124	117
Leeds	188	223	189	187	162	136	126
Leicester	*193*	*190*	*173*	*168*	140	108	102
Liverpool	301	346	286	303	254	235	212
Manchester	*274*	*290*	*243*	*242*	213	184	178
Newcastle	195	211	187	191	153	124	113
Norwich	*169*	*159*	*145*	*142*	111	97	87
Nottingham	*214*	*188*	*187*	*161*	136	129	98
Salford	*236*	*227*	*199*	*202*	196	167	163
Sheffield	196	225	213	205	184	148	145
Sunderland	169	186	184	158	158	137	127
Wolverhampton	191	221	218	179	140	118	119

*Notes*: The Registrar‐General reported deaths for the seven years 1838–44, and births for the six years 1839–44. We estimated the births in 1838 as 0.75 of the average births in 1838–44, to take account of population growth. This was a conservative estimate and probably biased estimates of infant mortality upward, and life expectancy downward.

*Sources: 8th–11th Annual Reports of the Registrar‐General* (P.P. 1847/8, XXV; 1849, XXI; 1850, XX); Woods, ‘Causes of death’.

**Figure 5:**
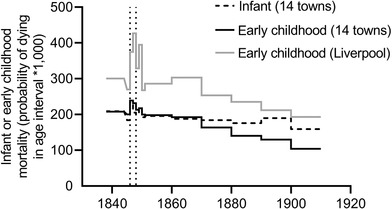
Infant and early childhood mortality in core registration districts of 14 large towns (unweighted means), and early childhood mortality in Liverpool, 1838–1910 *Note*: Vertical dashed lines indicate the years 1846–7 (see section III). *Sources*: See tab. 2.

The unweighted means of these series (for all 14 towns) are presented in figure 5, together with early childhood mortality rates for Liverpool. All series show the devastating effects of both the Irish famine and the cholera epidemic of 1849. Irish refugees flooded into Liverpool (and other mainly northern towns to a lesser extent) in 1846–7, and severely distorted mortality statistics for the town, first because many of them were desperately ill on arrival, and second because they had dispersed to a great extent by 1851, and so were not included in the denominator of mortality rates (which was estimated for intercensal years by interpolation between population counts in 1841 and 1851). The 1849 cholera epidemic caused high mortality (exceeding five deaths per 1,000 inhabitants) in Hull, Wolverhampton, Leeds, and Liverpool (and London), but was muted in most textile towns, and negligible in Birmingham.50
Davenport et al., ‘Cholera’.


To summarize these patterns, first, there was mixed evidence of heightened child mortality specifically in the period 1838–50, but to the extent that this was a real phenomenon (rather than an effect of under‐recording of births in this period) then it was not confined to new or industrial towns. Second, mortality was fairly stable across the period 1838–70. That is, there was little evidence of a crisis in mortality in the 1830s and 1840s that then abated in the later part of the 1850s, except in those towns most severely affected by the Irish famine. Rather the high mortality rates evident in the earliest period of civil recording were in the main sustained until 1870.

We could also calculate life expectancy at birth in the core registration districts of the largest towns for the period 1838–44, using the Registrar‐General's reports of births, deaths, and population by age for this period (table 3). Our estimates for 1838–44 are very much in line with Szreter and Mooney's calculations of life expectancies for the same towns in the 1850s and 1860s, and suggest fairly stable mortality levels across the period 1838–70, consistent with our analysis of mortality in the first five years of life.

**Table 3:** Life expectancy at birth in core registration districts of selected large towns, 1838–1900

Town	1838–44	1851–60	1861–70	1871–80	1881–90	1891–1900
Birmingham	36	35	35	37	39	38
Bradford	38	37	36	38	42	44
Bristol	33	35	36	37	39	43
Liverpool	27	27	25	28	29	30
Manchester	28	30	29	32	35	36
Newcastle	35	34	34	37	40	42
Sheffield	35	34	33	35	38	39
Unweighted mean	33	33	33	35	37	39

*Notes*: Life expectancy values for 1838–44 were calculated using abridged life tables based on five‐year age groups for ages 5–24 and 10‐year age groups for ages 25 and above. See notes to tab. 2 regarding counts of births. Those dying in infancy were assumed to have lived on average 0.3 of a year, and those surviving to 85, 3 years. Values for 1851–1900 are those reported by Szreter and Mooney.

*Sources: 8th and 9th Annual Reports of the Registrar‐General* (P.P. 1847/8, XXV) (values for 1838–44); Szreter and Mooney, ‘Urbanisation’, tab. 2, p. 90.

In contrast to our own estimates, Szreter and Mooney reported strikingly low values of life expectancy for Liverpool and Manchester in *c*. 1841 (of 25.7 and 25.3 years respectively), and these informed their very low estimates of life expectancy in large towns in this period (figure 2c).51
Szreter and Mooney, ‘Urbanisation’, p. 90. What is the cause of the discrepancy? In the case of Liverpool Szreter and Mooney reported the Registrar‐General's estimate from his life table for Liverpool for the year 1841.52
*5th Annual Report of the Registrar‐General* (P.P. 1843, XXI), pp. 33–6. This life table was based on deaths in a single year (1841), whereas our estimate in table 3 relies on deaths for the seven years 1838–44, and is probably a more representative average of the mortality experience of Liverpool in this period. In the case of Manchester, Szreter and Mooney again used the Registrar‐General's life table for Manchester but in this case the Registrar‐General used deaths for the years 1838–44, as we did. The key difference between our estimate and his was that he also had available to him deaths and population counts for the registration *sub*‐districts of Manchester, and he based his life table for Manchester on the sub‐districts that he considered urban. That is, his life expectancy estimate was based on the registration sub‐districts of Ancoats, Deansgate, St George, London Road, and Market‐street, with a population of 163,561, not the whole registration district (with a population of 192,403).53The Registrar‐General considered the sub‐districts of Blackley, Cheetham, Failsworth, Newton, and Prestwich to be rural. The population of 163,561 persons in the urban sub‐districts refers to those with stated ages; *7th Annual Report of the Registrar‐General* (P.P. 1846, vol. XIX), p. 330. The largest towns usually included one registration district that was largely urban, and sometimes additional districts that were partly or mainly rural but contained some fraction of the city population. There were very large differences in life expectancy between the ‘core’ urban registration districts and the more rural components, of three to 11 years in the 1850s.54
Szreter and Mooney, ‘Urbanisation’, p. 90. The differences were probably much larger in the case of Manchester registration sub‐districts, because the rural sub‐districts did not include any parts of the city in 1841. The crude death rate in the urban sub‐districts was 35 per 1,000 inhabitants in 1837–44, compared with 21 in the rural sub‐districts. In the case of Szreter and Mooney's estimate for Manchester in 1838–44, we would expect that the urban sub‐districts within Manchester registration district would be similarly disadvantaged with respect to the more rural sub‐districts, and therefore any estimate based on them would be lower than for the registration district as a whole, and would not be comparable with values for the registration district of Manchester in later decades.

Our estimates of life expectancy in the core registration districts of both Liverpool and Manchester and five other major cities in the period 1838–44 suggest that life expectancy in large cities did not change markedly between the beginning of registration in 1838 and the 1860s (as indicated by the unweighted mean for the sample as a whole, of 33 years across this period) (table 3).

## IV

The evidence presented in section II supported the scenario of a significant rise in early childhood mortality in England in the middle decades of the nineteenth century. This rise occurred not only in rapidly growing industrializing villages, but also in older towns and even rural populations, and was sustained from the late 1830s to the 1860s. However, we could not establish whether mortality also rose in the early decades of the nineteenth century in the largest English towns, because we had no data for these towns before 1838 (section III). In this section we take a comparative approach, to ask whether urban mortality rose elsewhere in the early nineteenth century. Where possible we focus on disaggregated urban and rural data, since aggregated national rates may reflect processes that occurred largely in towns. Unfortunately the first half of the nineteenth century was also a rather bleak period for urban demographic data in Europe and North America. Nevertheless the data that do exist for European and American cities suggest that the pattern of worsening mortality argued for England was a widespread phenomenon.

In the US crude death rates rose in the second quarter of the nineteenth century in New York, Boston, New Orleans, and Philadelphia, but not apparently in Baltimore.55Haines, ‘Urban mortality’. See also Condran, ‘New York’; Vinovskis, ‘Massachusetts’. The timing of the rise differed somewhat by city, being restricted mainly to the period 1820–40 in Philadelphia, but later in the other cities. These five eastern seaboard cities all grew rapidly in the first half of the nineteenth century. Haines commented that ‘one must conclude that large American cities had become virtual charnel houses by the middle of the xixth century’.56Haines, ‘Urban mortality’, p. 37. Haines attributed these rises to ‘rapid [urban] population growth, combined with large numbers of immigrants and the increased movement of goods and people’ that overwhelmed early attempts to improve sanitation and prevent disease transmission.57Ibid., pp. 44–5.


Haines did not make any comparison between the eighteenth and nineteenth centuries, because of the general paucity of data for American cities before the nineteenth century. However, what estimates do exist suggest that despite their relative isolation and comparatively small populations, American cities were probably more lethal in the eighteenth century than the nineteenth. Blake estimated that the crude death rate in Boston rarely dipped below 30 per 1,000 in the period 1701–74, whereas it only exceeded this rate three times in the nineteenth century.58
Blake, *Public health*, pp. 247–50. Klepp estimated that crude death rates in Philadelphia averaged 36 per 1,000 in the period 1788–1801, a level reached only twice in the nineteenth century.59
Klepp, *Swift progress*, p. 217. See Smith, ‘Death and life’, for even higher crude death rates estimates for the period 1725–75. Therefore, although the middle decades of the nineteenth century appear to have witnessed a rise in mortality in some US cities, it is unlikely that these trends represented an unprecedented worsening of urban health.60The apparent lethality of relatively small port towns in colonial America was only in small part a function of occasional yellow fever outbreaks. See, for example, Blake, *Public health*, p. 99; Anroman, ‘Philadelphia’. The high background levels of mortality, like those of European towns in the same period, require another explanation.


**Figure 6:**
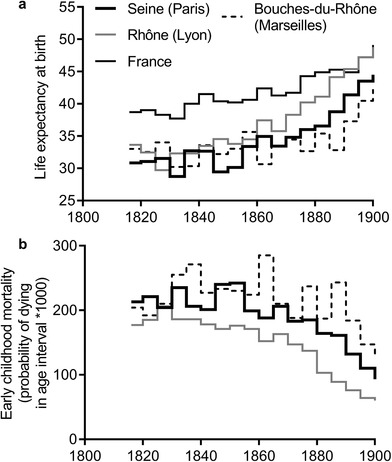
Female life expectancy (panel a) and early childhood mortality (ages 1–4 years) (panel b) in French departments *Source*: Preston and van der Walle, ‘French mortality’, p. 277.

Preston and van der Walle produced estimates of female age‐specific mortality and life expectancy for French departments containing the largest French cities of Paris, Lyon, and Marseilles, between 1816 and 1905 (figure 6a). In the case of Paris and Marseilles life expectancy fell in the second quarter of the nineteenth century. In Lyon there was a slight drop in life expectancy in the period 1825–34, but the pattern was largely one of little change between 1816 and 1850.61But see n. 62. Examining age‐specific rates, it is clear that the worsening of mortality in the departments that included Paris and Marseilles was a consequence largely of rises in mortality in early childhood, at one to four years of age, but not in infancy (figure 6b).62
Preston and van der Walle, ‘French mortality’, pp. 277, 285–6. The authors attributed the divergence between Rhône (Lyon) and the other departments to superior water supply and sanitary provision in the former. However the poor quality of the data for Rhône made it necessary to use model life tables to estimate mortality at ages under five years in this department. The procedure adopted meant that trends in under‐five mortality were constrained to follow those of older age groups, a method that would obscure any age‐specific rises in mortality among children. Indeed the authors noted that no single model life table could be made to fit the full age range, and that different model life tables were appropriate for early age mortality and adult mortality in Seine and Bouches‐du‐Rhône. All three cities grew rapidly over the first half of the nineteenth century. Paris almost doubled in population, from roughly 580,000 to over one million, between 1800 and 1850, while both Marseilles and Lyon grew by roughly 60 per cent (from 111,000 to 193,300 and from 110,000 to 177,200 respectively).63Lahmeyer, ‘France’.


**Figure 7:**
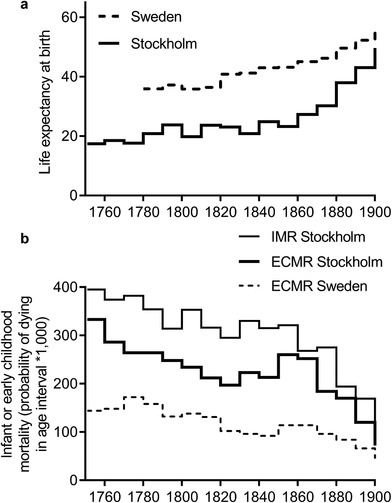
Panel a: life expectancy at birth in Stockholm and the whole of Sweden; panel b: mortality in infancy (IMR) in Stockholm and mortality in early childhood (ECMR) in Stockholm and the whole of Sweden *Source*: Woods, ‘Historical relationship’, app. tab. 2.

In Sweden an increase in the volatility of mortality patterns in the middle decades of the nineteenth century has been described as the ‘last manifestation of the great epidemic cycles which characterised pre‐industrial society’, after a period of rapid mortality decline and stabilization from the late eighteenth century.64
Fridlizius, ‘Malmö’, p. 125. This phenomenon was most marked in Swedish towns. In Stockholm mortality rose in the 1840s, and this was a function largely of a pronounced worsening of mortality rates in the one‐to‐four‐years age group (figure 7a and b). This rise in mortality among young children was common to towns and rural populations of Sweden in the mid‐nineteenth century, but was most pronounced in the port towns of Stockholm and Malmö.65Ibid., pp. 128, 141. Stockholm is particularly interesting because unlike the other cities included here Stockholm experienced little economic or demographic growth in the first half of the nineteenth century.66
Söderberg et al., *Stagnating metropolis*. A similar rise in childhood mortality occurred in the Danish population in the 1850s and 1860s, in both rural and urban populations.67
Johansen, *Danish population history*, pp. 144, 147.


In eastern Belgium Alter et al. have described an ‘epidemiological depression’ affecting most of the nineteenth century. They attributed this to the ecological effects of rapid industrialization, and the assortment of the regional population into rapidly growing industrial and mining districts. Although direct evidence is lacking for towns in the first half of the nineteenth century, it is very likely that mortality rose markedly in many of the settlements that grew into towns in this period. A general worsening of conditions is presumed on the basis of the very high mortality rates evident in both new and older towns in the period *c*. 1850–70. Longer time series exist for the rural areas of Sart and Polleur, and here life expectancy improved in the first decades of the nineteenth century, but fell again in the period 1850–70.68Alter, Neven, and Oris, ‘Sart’, p. 182; Neven, ‘East Belgium’, p. 46; idem, ‘Tilleur’.


A rise in crude death rates in the middle decades of the nineteenth century is also evident in St Petersburg and Russia and in the largest Prussian towns.69
Imhof, ‘Methodological problems’, p. 103; Kearns et al., ‘Interaction’, p. 12; Wheatcroft, ‘Eastern Europe’, p. 222. In the Netherlands post‐neonatal and early childhood mortality rose in the middle decades of the nineteenth century in the mainly rural provinces of Zeeland and Friesland, and more markedly in the more urban province of Utrecht. Estimates of life expectancy suggest a pronounced downturn in the same period in Amsterdam.70
van Leeuwen and Oeppen, ‘Amsterdam’; van Poppel, Jonker, and Mandemakers, ‘Three Dutch regions’. In northern Italy life expectancy fell in the rural villages of Casalguidi and Madregolo and their associated regions (Tuscany and the Duchy of Modena and Reggio respectively) in the 1850s. These falls followed probable improvements in these areas in the 1820s–40s.71Breschi, Derosas, and Manfredini, ‘Mortality’, pp. 216–20. However this pattern of worsening mortality rates in the mid‐nineteenth century was not observed everywhere. In the Canadian province of Quebec crude death rates, life expectancy, and infant mortality appear to have improved across the nineteenth century in both rural and urban populations, despite the very rapid growth of the towns of Quebec City and Montreal in this period (although life expectancy at the age of 10 years fell briefly in the 1830s).72Pelletier, Légaré, and Bourbeau, ‘Quebec’.


The broad apparent similarity in trends in mortality in cities of the US Atlantic seaboard, France, Britain, and Scandinavia supports the argument that mortality worsened in urban centres in the second quarter of the nineteenth century in Europe and North America. Evidence from rural Scandinavia, northern Italy, and possibly Belgium and the Netherlands suggests that early childhood mortality also worsened in rural populations, and this is consistent with the evidence presented in section II that mortality rose in rural as well as urban and industrializing settlements in England. This raises the question of whether these trends were driven by common phenomena. The case of Stockholm and of rural populations in Scandinavia and England suggests that, to the extent that international trends were driven by the same factors, then factors other than rapid population growth or unbridled economic development may have played a role.

## V

The evidence for elevated mortality in the mid‐nineteenth century in rural populations and in older and slower‐growing towns as well as rapidly growing settlements suggests that other influences were operating in addition to any worsening of urban living conditions. In the case of the US, Haines et al. argued that mortality rose in rural as well as urban centres as the former were drawn into national markets, and rural populations were increasingly exposed to infectious diseases and denied the supposed nutritional benefits of subsistence farming.73Haines, Craig, and Weiss, ‘“Antebellum puzzle”’. A similar process of epidemiological integration has been evoked to account for the relative high mortality of even small market towns and rural populations in England in the period *c*. 1650–1750; however, these processes were probably largely complete by the early nineteenth century.74See n. 7. Here we evaluate an exogenous factor that has been widely recognized as a major influence on mortality patterns in this period, and that could have produced the specific phenomenon observed, of a very widespread and roughly synchronous rise in early childhood mortality. This is the apparent change in the biological properties of the causal agent of scarlet fever.

**Figure 8:**
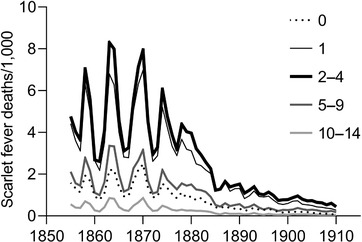
Annual scarlet fever mortality by age group, England and Wales, 1855–1911 *Notes*: Deaths attributed to scarlet fever were reported together with diphtheria in the annual returns of the Registrar‐General until 1858, but diphtheria deaths were reported separately in supplementary tables from 1855, and have been subtracted from reported totals for these years. *Sources*: Davenport, ‘Annual deaths’.

Scarlet fever had its greatest impact in early childhood (figure 8). Mortality attributed to the disease bore an apparently sigmoidal relationship to population density (figure 4d), and scarlet fever was an important cause of death in rural as well as urban populations. It was the leading cause of death at ages one to nine years in the English population by the mid‐nineteenth century, when national records became available, and accounted for much of the anomalously high rates of early childhood mortality, relative to infant mortality, in this period (table 4).75
Woods, ‘Historical relationship’, p. 215; Woods and Shelton, *Atlas*, p. 76. As figure 8 indicates, it was the cause of the major epidemic spikes in mortality evident at ages one to nine years between 1840 and 1870 (figure 2b). Its decline from the 1870s was a major driver of the early mortality decline among young children, and therefore made a very substantial contribution to the secular rise in life expectancy from the 1870s onwards.76
Hinde and Harris, ‘Mortality decline’; Kearns, ‘Le handicap urbain’; McKeown and Record, ‘Reasons’, pp. 346–59; Woods, *Demography*, pp. 323–4, 359.


**Table 4:** Deaths attributed to scarlet fever as a proportion of all deaths at ages 1–4 years

Population	Period	% of deaths aged 1–4
Manchester	1830–44	7.24
Glasgow	1836–42	7.08
Perth	1837–41	8.64
England and Wales	1851–60	13.3
England and Wales	1861–70	14.8
England and Wales	1871–80	13.1
England and Wales	1881–90	3.6
England and Wales	1891–1900	2.0
England and Wales	1901–10	2.0

*Notes*: Annual scarlet fever deaths were reported together with diphtheria until 1855 in England and Wales (see fig. 8); however, the number of deaths attributed to diphtheria appears to have been very small before the 1860s, both in England and Wales and in Philadelphia. The creation of a separate category for diphtheria did not appreciably reduce the numbers of scarlet fever deaths; Condran, ‘Diphtheria’; Creighton, *History*, p. 614 and ch. 7. Deaths attributed to scarlet fever and diphtheria were reported separately in the decennial returns for England and Wales in all decades (1851–1910).

*Sources*: For Manchester: Manchester Archive, GB127.M74/3/15/1‐3, Rusholme Rd cemetery burials, 1830–49; for Perth: Sykes, Sandon, Porter, Heywood, Alison, and Chadwick, *Report*, pp. 164–8; for Glasgow: Watt, *Glasgow bills*, pp. 48–59; for England and Wales: Woods, *Causes of death*.

**Figure 9:**
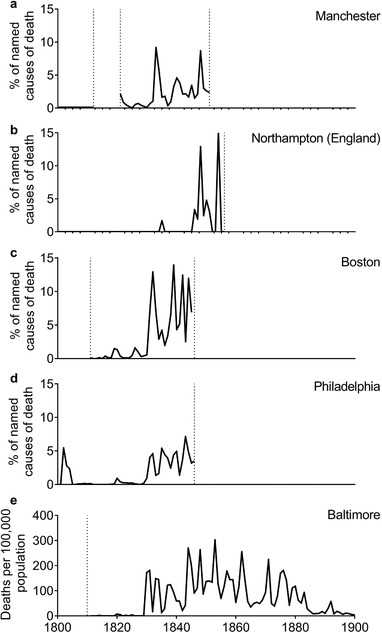
Scarlet fever as a percentage of all burials with given cause (panels a, b, c, and d) or deaths per 100,000 population (panel e) *Notes*: Dashed lines indicate periods with cause of death data: series in panels a, b, and d extend from 1800. *Sources*: Panel a: Manchester Cathedral Archives, MS18/1–10, sextons’ burial books of the collegiate church, Manchester; Manchester Central Library, Manchester and Lancashire Family History Society, MFPR 1118,1142, St John Deansgate burial registers; Manchester Archive, GB127.M74/3/15/1‐3, Rusholme Rd cemetery burials, 1830–49. Panel b: BL, 1877.e.2–3, All Saint's parish, Northampton bills of mortality. Panel c: Shattuck, *Report*, pp. 76–9. Panel d: Klepp, *Swift progress*, pp. 61–284. Panel e: Howard, *Public health*, ch. 11.

Table 4 includes cause of death data for Manchester, Glasgow, and Perth derived from local sources. Cause of death data are fairly sparse before *c*. 1850, and for Britain we are restricted largely to data derived from the London and Glasgow bills of mortality and to burial records from individual urban parishes and cemeteries within larger urban units. The latter sources in particular provide cross‐tabulation of deaths by cause and age. We have also collated or transcribed cause of death data for three American cities (Baltimore, Boston, and Philadelphia) (figure 9).

The disease recognized as scarlet fever is caused by toxins produced during infection with strains of group A beta‐haemolytic streptococci (*Streptococcus pyogenes*).77Streptoccoci were implicated in scarlet fever infection by Edward Klein in 1887, and the aetiology of the disease and the role of toxins in producing symptoms were elucidated by Gladys Dick and George Dick in the 1920s; Hardy, ‘Scarlet fever’, Dick and Dick, ‘Etiology’. Streptococci are well‐known for wide variations in virulence, and it is broadly accepted among demographic and medical historians that the streptococcal pathogens causing scarlet fever underwent some kind of change in virulence in the 1830s.78
Hardy, *Epidemic streets*, p. 59; Katz and Morens, ‘Streptococcal infections’; Swedlund and Donta, ‘Scarlet fever’. As the great nineteenth‐century compiler of disease statistics, Charles Creighton, observed, ‘The enormous number of deaths from scarlatina during some thirty or forty years in the middle of the nineteenth century will appear in the history as one of the most remarkable things in our epidemiology’.79
Creighton, *History*, p. 726.


The apparently explosive rise in scarlet fever deaths in the 1830s is evident in cities in Britain and the US (figure 9). The dotted lines in figure 9 indicate the start and finish of each cause of death series. A similar rise appears to have occurred in the 1840s or 1850s in Sweden and Denmark.80
Fridlizius, ‘Malmö’, pp. 143–6; Johansen, *Danish population history*, fig. 5.8b, pp. 144–5. The remarkable rise in scarlet fever mortality in the mid‐nineteenth century at first glance suggests some abrupt change in recording practices. However, this does not seem to be the explanation. Scarlet fever *was* probably under‐recorded in the London bills of mortality, where it was subsumed under ‘fever’ or ‘measles’ until 1830. Creighton described a number of eighteenth‐century epidemics of ‘sore‐throat’ that included the typical symptoms of scarlet fever and some of its more unusual epidemiological characteristics, in particular pronounced autumnal peaks and greatest mortality at two to seven years of age. However scarlet fever, or scarlatina, was described by Sydenham in the late seventeenth century, and was widely recognized as a distinct disease by the early nineteenth century.81
Creighton, *History*, pp. 680, 706–24. In Britain medical case notes and dispensary records recorded large numbers of scarlet fever cases in the first three decades of the nineteenth century; however, it seems that fatalities from the disease were rare. Contemporary observers certainly noted the change in the nature of the disease after 1830.82Ibid., pp. 722–7. The near‐simultaneous eruption of scarlet fever epidemics in port cities in Scandinavia, the US, and Britain suggests the emergence of a distinct epidemiological phenomenon. With respect to Sweden, Fridlizius remarked on the apparently sequential spread of scarlet fever from port cities in western Sweden to other towns and eventually into rural areas by the 1860s.83
Fridlizius, ‘Malmö’, p. 145. A similar pattern, consistent with the introduction of a new disease strain and its subsequent dissemination within urban systems, is suggested by the lag between the appearance of epidemic scarlet fever in Copenhagen in the 1840s and in provincial Danish towns in the 1860s, and in London and Manchester in the 1830s and, belatedly, in Northampton in the 1840s (figure 9).84
Johansen, *Danish population history*, fig. 5.8b, p. 145. Such lags are also consistent with the variations in the timing of the mid‐century rise in mortality, documented in section IV.


The disease waned again from around 1870 (figure 8). In England scarlet fever mortality declined by 90 per cent between the 1860s and 1901–10 in all registration districts regardless of population density.85
Hinde and Harris, ‘Mortality decline’, pp. 10–16. Case‐fatality records from the last quarter of the nineteenth century, when scarlet fever was already declining in importance, indicate falling rates of deaths per infection (that is, virulence) in London, Norway, and Baltimore.86
Backer, *Trends*, p. 82; Howard, *Public health*, p. 310; Katz and Morens, ‘Streptococcal infections’.


The synchronized rise and fall of scarlet fever as a major cause of early childhood mortality between *c*. 1830 and *c*. 1870, in populations across Europe and North America, suggest very strongly that this was an autonomous biological phenomenon driven by changes in the virulence of the pathogen, albeit one that was propagated by networks of communication between international ports, and between ports and their hinterlands. This phenomenon was not limited to industrial cities, and appears to have been driven predominantly by biological rather than social or economic factors, although the latter probably played a crucial role in the dissemination of novel pathogen strains.

## VI

Following dramatic improvements in urban survival rates in the late eighteenth and early nineteenth centuries, there appear to have been reversals to this trend between *c*. 1830 and the 1860s. In the case of Britain, mortality increases especially in early childhood were sufficient to bring a halt to secular improvements in life expectancy at the national level. This stagnation in mortality improvements has led to the perception that the demographic transition only got underway in Britain from the 1870s. However, it is likely that without prior improvements in urban mortality, and without countervailing trends that prevented urban mortality rates from rising again to their eighteenth‐century levels, then the rapid urbanization of the nineteenth century in Britain would have resulted in substantial *falls* in national life expectancy. The impact of urbanization on health in the first half of the nineteenth century is of particular interest to historians because of its relevance to the long‐running ‘standards of living’ debate, regarding the early impacts of industrialization on the British population. Szreter and Mooney's work provides the key reference for claims regarding deteriorations in urban health in this period, and we suggest that their views require some qualification.87Szreter and Mooney, ‘Urbanisation’. These qualifications relate to the specificity, timing, and magnitude of any worsening in mortality, and imply that any downturn in life expectancy in this period was not simply a corollary of rapid economic development.

First, the worsening of urban mortality rates in the middle decades of the nineteenth century was not confined to British industrial towns and cities. Rather it appears to have been a very widespread phenomenon that affected rural as well as urban populations. The apparent ubiquity of this phenomenon supports Woods's argument that administrative collapse and laissez‐faire economic policy are unnecessary to explain the mortality patterns of this period.88Woods, *Demography*, pp. 268–71. Elsewhere we have shown that English industrial and manufacturing towns were not at especial risk of waterborne diseases.89
Davenport et al., ‘Cholera’. It is therefore very likely that mortality would have risen in towns in this period even in the absence of economic growth or urbanization (as was broadly the case in Sweden).

Second, our evidence indicates that any rises in mortality in the middle decades of the nineteenth century were not confined to the period *c*. 1830–50. We found no evidence, for the 14 largest towns or for the reconstitution sample parishes and their registration districts, that mortality fell consistently in infancy or early childhood in the 1850s. Instead the major falls occurred after 1870, consistent with the existing historiography of early childhood mortality and with patterns of scarlet fever mortality. The sudden rise and more gradual fall of childhood mortality between the 1830s and the end of the nineteenth century matches remarkably closely the rise and fall of scarlet fever as a major cause of childhood mortality in England and Wales. It is widely accepted that scarlet fever underwent an autonomous increase in virulence in the early nineteenth century, that then waned again after *c*. 1870.90See nn. 75 and 78. This article is, however, the first that we know of to draw together the existing comparative evidence for scarlet fever trends in the first half of the nineteenth century. The suddenness and scale of the scarlet fever epidemics that erupted in cities in Britain, Scandinavia, and the US in the 1830s provide a plausible explanation for the otherwise very puzzling abruptness, and age‐specificity, of the rises in childhood mortality in the English and Swedish populations in this period (figures 2b, 3, and 7; tables 1 and 3). Scarlet fever was the leading cause of death in early childhood in England in the 1850s and 1860s, and a decline in scarlet fever virulence is widely accepted as the main reason for the precocious decline in early childhood mortality after *c*. 1870.91See n. 76. In the absence of reliable cause of death data for defined populations before the late 1840s, we cannot quantify fully the contribution of scarlet fever to wider mortality trends, and it is likely that other factors, including the Irish famine and the epidemiological effects of rising population densities and connectedness, influenced national patterns. However, scarlet fever should be considered a major explanatory factor.

Our evidence indicates that there were indeed very widespread rises especially in early childhood mortality in the second quarter of the nineteenth century, that were sustained until roughly 1870. The ubiquity of these patterns suggests that they were not straightforwardly a function of urbanization or industrialization. Nonetheless, any worsening of mortality in towns would have exacerbated the trade‐offs between health and wealth entailed by migration to towns, and depressed the ‘biological standard of living’. Moreover, to the extent that early childhood mortality in the period 1830–70 reflected heightened exposure to infectious diseases, then such exposure could have imposed higher energetic demands on the survivors, or produced long‐lasting ill health, that resulted in sustained stunting.92Increased infectious disease mortality could have resulted from increased exposure to disease, or from increased susceptibility, for example, as a result of poor nutrition. However, in the case of scarlet fever at least an increase in exposure can be assumed. Anthropometric evidence from military recruits and prisoners in Britain and the US suggests that adult stature was reduced in cohorts born in the middle quarters of the nineteenth century.93Floud, Wachter, and Gregory, *Height, health and history*, pp. 148–9; Fogel, ‘Nutrition’. Our results therefore lend tentative support to arguments that childhood health and development were depressed in the mid‐nineteenth century. However, it remains very unclear whether the observed hikes in childhood mortality, especially from scarlet fever, were associated with increases in chronic ill health, or represented acute events with few sequelae for survivors.94Notably, French and Swedish conscripts displayed roughly monotonic rises in stature across the nineteenth century, despite experiencing similar rises in childhood mortality in the mid‐nineteenth century; Sandberg and Steckel, ‘Sweden’; Weir, ‘Economic welfare’. See also Bodenhorn, Guinnane, and Mroz, ‘Sample‐selection biases’.


Notwithstanding the deterioration of survival rates in the mid‐nineteenth century, mortality in British towns was much lower in the nineteenth century than in the eighteenth. Therefore any attempt to relate the health disamenities of towns directly to the disruptive impacts of industrialization must acknowledge the enormous improvements in urban mortality rates that appear to have accompanied early industrialization, and which were not completely reversed even by unprecedented rates of urbanization in the nineteenth century. When placed in an international and long‐run demographic context, the ‘new’ industrial and manufacturing cities of the British industrial revolution did not represent a nadir in urban health. This is not to diminish in any way the appalling conditions that prevailed in these cities, but simply to draw attention to the enormous and ubiquitous dangers associated with urban life generally before the late nineteenth century, some of which were exogenous, in the narrow sense, to economic conditions.

Footnote references

Alsan, M.
 and 
Goldin, C.
, ‘Watersheds in child mortality: the role of effective water and sewerage infrastructure, 1880 to 1920’, Journal of Political Economy, 127 (2019), pp. 586–638.3107324910.1086/700766PMC6502471

Alter, G.
, 
Neven, M.
, and 
Oris, M.
, ‘Mortality and modernization in Sart and surroundings, 1812–1900’, in BengtssonT., CampbellC., and LeeJ. Z., eds., Life under pressure: mortality and living standards in Europe and Asia, 1700–1900 (2004), pp. 173–208.

Anroman, G. M.
, ‘Infectious disease in Philadelphia, 1690–1807: an ecological perspective’, in ZuckermanM. K., ed., Modern environments and human health: revisiting the second epidemiologic transition (Hoboken, NJ, 2014), pp. 17–34.

Armstrong, W. A.
, ‘The trend of mortality in Carlisle between the 1780s and the 1840s: a demographic contribution to the standard of living debate’, Economic History Review, 2nd ser., XXXIV (1981), pp. 94–114.10.1111/j.1468-0289.1981.tb02008.x11614426

Backer, J. E.
, Trends of mortality and causes of death in Norway 1856–1955 (Oslo, 1961).

Bennett, R. J.
, ‘Urban population database, 1801–1911’, (2012), UK Data Service, SN: 7154, 10.5255/UKDA-SN-7154-1.

Blake, J. B.
, Public health in the town of Boston 1630–1822 (Cambridge, Mass., 1959).

Bodenhorn, H.
, 
Guinnane, T. W.
, and 
Mroz, T. A.
, ‘Sample‐selection biases and the industrialization puzzle’, Journal of Economic History, 77 (2017), pp. 171–207.

Breschi, M.
, 
Derosas, R.
, and 
Manfredini, M.
, ‘Mortality and environment in three Emilian, Tuscan, and Venetian communities, 1800–1883’, in BengtssonT., CampbellC., and LeeJ. Z., eds., Life under pressure: mortality and living standards in Europe and Asia, 1700–1900 (2004), pp. 209–52.

Carmichael, A. G.
 and 
Silverstein, A. M.
, ‘Smallpox in Europe before the seventeenth century: virulent killer or benign disease?’, Journal of the History of Medicine and Allied Sciences, 42 (1987), pp. 147–68.329502610.1093/jhmas/42.2.147

Chapman, J.
, ‘The contribution of infrastructure investment to Britain's urban mortality decline, 1861–1900’, Economic History Review, 72 (2019), pp. 233–59.

Condran, G. A.
, ‘Changing patterns of epidemic disease in New York City’, in RosnerD., ed., Hives of sickness: public health and epidemics in New York City (New Brunswick, NJ, 1995), pp. 27–41.

Condran, G. A.
, ‘The elusive role of scientific medicine in mortality decline: diphtheria in nineteenth‐ and early twentieth‐century Philadelphia’, Journal of the History of Medicine and Allied Sciences, 63 (2008), pp. 484–522.1853961310.1093/jhmas/jrn039

Creighton, C.
, A history of epidemics in Britain, vol. II (Cambridge, 1894).

Cutler, D.
 and 
Miller, G.
, ‘The role of public health improvements in health advances: the twentieth‐century United States’, Demography, 42(1) (2005), pp. 1–22.1578289310.1353/dem.2005.0002

Davenport, R. J.
, ‘Annual deaths by cause, age and sex in England and Wales, 1848–1900’, UK Data Service, SN: 5705 (2007).

Davenport, R. J.
 ‘Infant‐feeding practices and infant survival by familial wealth in London, 1752–1812’, History of the Family, 24 (2019), pp. 174–206.3105827210.1080/1081602X.2019.1580601PMC6474727

Davenport, R. J.
, 
Boulton, J. P.
, and 
Schwarz, L.
 ‘The decline of adult smallpox in eighteenth‐century London’, Economic History Review, 64 (2011), pp. 1289–314.2217140410.1111/j.1468-0289.2011.00599.xPMC4373148

Davenport, R. J.
, 
Boulton, J. P.
, and 
Schwarz, L.
, ‘Urban inoculation and the decline of smallpox in eighteenth‐century cities—a reply to Razzell’, Economic History Review, 69 (2016), pp. 188–214.2690016910.1111/ehr.12112PMC4737216

Davenport, R. J.
, 
Satchell, M.
, and 
Shaw‐Taylor, L. M. W.
, ‘The geography of smallpox in England before vaccination: a conundrum resolved’, Social Science and Medicine, 206 (2018), pp. 75–85.2968465110.1016/j.socscimed.2018.04.019PMC5958952

Davenport, R. J.
, 
Satchell, M.
, and 
Shaw‐Taylor, L. M. W.
, ‘Cholera as a “sanitary test” of British cities, 1831–1866’, History of the Family, 24 (2019), pp. 403–38.10.1080/1081602X.2018.1525755PMC658245831274973

Dick, G. F.
 and 
Dick, G. H.
, ‘The etiology of scarlet fever’, Journal of the American Medical Association, 82 (1924), pp. 301–2.

Duggan, A. T.
, 
Perdomo, M. F.
, 
Piombino‐Mascali, D.
, 
Marciniak, S.
, 
Poinar, D.
, 
Emery, M. V.
, 
Buchmann, J. P.
, 
Duchêne, S.
, 
Jankauskas, R.
, 
Humphreys, M.
, 
Golding, G. B.
, 
Southon, J.
, 
Devault, A.
, 
Rouillard, J. M.
, 
Sahl, J. W.
, 
Dutour, O.
, 
Hedman, K.
, 
Sajantila, A.
, 
Smith, G. L.
, 
Holmes, E. C.
, 
Poinar, H. N.
, ‘17th century variola virus reveals the recent history of smallpox’, Current Biology, 26 (2016), pp. 3407–12.2793931410.1016/j.cub.2016.10.061PMC5196022

Floris, J.
 and 
Staub, K.
, ‘Water, sanitation and mortality in Swiss towns in the context of urban renewal in the late nineteenth century’, History of the Family, 24 (2019), pp. 249–76.

Floud, R.
, 
Wachter, K.
, and 
Gregory, A.
, Height, health and history. Nutritional status in the United Kingdom, 1750–1980 (Cambridge, 1990).

Fogel, R.W.
, ‘Nutrition and the decline in mortality since 1700: some preliminary findings’, in EngermanS. L. and GallmanR. E., eds., Long‐term factors in American economic growth (Chicago, Ill., 1986), pp. 439–555.

Fridlizius, G.
, ‘The mortality development of a port‐town in a national perspective: the experience of Malmö, Sweden, 1820–1914’, in LawtonR. and LeeR., eds., Population and society in western European port cities, c. 1650–1939 (Liverpool, 2002), pp. 124–75.

Galley, C.
, ‘A model of early modern urban demography’, Economic History Review, XLVIII (1995), pp. 448–69.

Galley, C.
, The demography of early modern towns: York in the sixteenth and seventeenth centuries (Liverpool, 1998).

Galley, C.
 and 
Shelton, N.
, ‘Bridging the gap: determining long‐term changes in infant mortality in pre‐registration England and Wales’, Population Studies, 55 (2001), pp. 65–77.

Haines, M. R.
, ‘The urban mortality transition in the United States, 1800–1940’, Annales de Demographie Historique, 101, (2001), pp. 33–64.

Haines, M. R.
, 
Craig, L. A.
, and 
Weiss, T.
, ‘The short and the dead: nutrition, mortality, and the “antebellum puzzle” in the United States’, Journal of Economic History, 63 (2003), pp. 382–413.

Hardy, A.
, The epidemic streets: infectious disease and the rise of preventative medicine 1856–1900 (Oxford, 1993).

Hardy, A.
 ‘Scarlet fever’, in KipleK. F., ed., The Cambridge historical dictionary of disease (Cambridge, 2003), pp. 288–90.

Harris, B.
 and 
Hinde, A.
, ‘Sanitary investment and the decline of urban mortality in England and Wales, 1817–1914’, History of the Family, 24 (2019), pp. 339–76.

Hinde, A.
 and 
Harris, B.
, ‘Mortality decline by cause in urban and rural England and Wales, 1851–1910’, History of the Family, 24 (2019), pp. 377–403.

Howard,
W. T.
, Public health administration and the natural history of disease in Baltimore, Maryland 1797–1920 (Washington, DC, 1924).

Huck, P.
, ‘Infant mortality in nine industrial parishes in northern England, 1813–36’, Population Studies, 48 (1994), pp. 513–26.1163945610.1080/0032472031000148006
*Human mortality database*
. University of California, Berkeley (US), and Max Planck Institute for Demographic Research (Germany), http://www.mortality.org (accessed on 8 July 2019).

Imhof, A. E.
, ‘Methodological problems in modern urban history writing: graphic representations of urban mortality 1750–1850’, in PorterR. and WearA., eds., Problems and methods in the history of medicine (1987), pp. 101–32.

Johansen, H. C.
, Danish population history 1600–1939 (Odense, 2002).

Katz, A. R.
 and 
Morens, D. M.
, ‘Severe streptococcal infections in historical perspective’, Clinical Infectious Diseases, 14 (1992), pp. 298–307.157144510.1093/clinids/14.1.298

Kearns, G.
, ‘Le handicap urbain et le déclin de la mortalité en Angleterre et au Pays de Galles, 1851–1900’, Annales de Démographie Historique (1993), pp. 75–105.11623388

Kearns, G.
, 
Lee, R. W.
, and 
Rogers, J.
, ‘The interaction of political and economic factors in the management of urban public health’, in NelsonM. C. and RogersJ., eds., Urbanisation and the epidemiologic transition (Uppsala, 1989), pp. 9–82.

Kitson, P.
, ‘Industrialization and the changing mortality environment in an English community during the industrial revolution’, in ZuckermanM. K., ed., Modern environments and human health: revisiting the second epidemiological transition (Hoboken, NJ, 2014), pp. 179–99.

Klepp, S. E.
, ‘The swift progress of population’: a documentary and bibliographic study of Philadelphia's growth, 1642–1859 (Philadelphia, Pa., 1991).

Lahmeyer, J.
, ‘France: historical demographical data of the administrative division: departments’, populstat.info/Europe/francep.htm (accessed on 13 June 2018).

Landers, J.
, ‘Historical epidemiology and the structural analysis of mortality’, Health Transition Review, 2, suppl. (1992), pp. 47–75.

Landers, J.
, Death and the metropolis: studies in the demographic history of London, 1670–1830 (Cambridge, 1993).

van Leeuwen, M. H. D.
 and 
Oeppen, J. E.
, ‘Reconstructing the demographic regime of Amsterdam 1681–1920’, Economic and Social History in the Netherlands, 5 (1993), pp. 61–102.

Levine, D.
, *Family formation in an age of nascent capitalism* (1977).

Luckin, B.
 and 
Mooney, G.
, ‘Urban history and historical epidemiology: the case of London, 1860–1920’, Urban History, 24 (1997), pp. 37–55.

McKeown, T.
 and 
Record, R. G.
, ‘Reasons for the decline of mortality in England and Wales during the nineteenth century’, Population Studies, 16 (1962), pp. 94–122.11630508

McNeill, W. H.
, ‘Migration patterns and infection in traditional societies’, in StanleyN. F. and JoskeR. A., eds., Changing disease patterns and human behaviour (1980), pp. 28–36.

Mercer, A.
, Disease, mortality and population in transition (Leicester, 1990).

Neven, M.
, ‘Epidemiology of town and countryside: mortality and causes of death in East Belgium, 1850–1910’, Revue Belge d'Histoire Contemporaine, 27 (1997), pp. 39–82.

Neven, M.
, ‘Mortality differentials and the peculiarities of mortality in an urban‐industrial population: a case study of Tilleur, Belgium’, Continuity and Change, 15 (2000), pp. 297–329.

Newton, G.
, ‘Infant mortality variations, feeding practices and social status in London between 1550 and 1750’, Social History of Medicine, 24 (2011), pp. 260–80.

Oris, M.
 and 
Fariñas
, 
D. R.
, ‘New approaches to death in cities during the health transition: an introduction’, in OrisM. and FariñasD. R., eds., New approaches to death in cities during the health transition (Geneva, 2016), pp. 1–16.

Pelletier, F.
, 
Légaré, J.
, and 
Bourbeau, R.
, ‘Mortality in Quebec during the nineteenth century: from the state to the cities’, Population Studies, 51 (1997), pp. 93–103.1161898810.1080/0032472031000149766

Peltola, J.
 and 
Saaritsa, S.
, ‘Later, smaller, better? Water infrastructure and infant mortality in Finnish cities and towns, 1870–1938’, History of the Family, 24 (2019), pp. 277–306.

van Poppel, F.
 and 
van der Heijden, C.
, ‘The effects of water supply on infant and childhood mortality: a review of historical evidence’, Health Transition Review, 7 (1997), pp. 113–48.10176376

van Poppel, F.
, 
Jonker, M.
, and 
Mandemakers, K.
, ‘Differential infant and child mortality in three Dutch regions, 1812–1909’, Economic History Review, LVIII (2005), pp. 272–309.

Preston, S. H.
 and 
van de Walle
, 
E.
, ‘Urban French mortality in the nineteenth century’, Population Studies, 32 (1978), pp. 275–97.11630579

Sandberg, L. G.
 and 
Steckel, R. H.
, ‘Was industrialization hazardous to your health? Not in Sweden!’, in SteckelR. H. and FloudR., eds., Health and welfare during industrialization (1997), pp. 127–60.

Sharlin, A.
, ‘Natural decrease in early modern cities: a reconsideration’, Past and Present, 79 (1978), pp. 126–38.

Sharpe, P.
, ‘Population and society 1700–1840’, in ClarkP., ed., Cambridge urban history of Britain, *II*: *1540–1840* (Cambridge, 2000), pp. 491–528.

Shattuck, L.
, Report to the committee of the city council appointed to obtain the census of Boston for the year 1845, embracing collateral facts and statistical researches (Boston, Mass., 1846).

Smith, B. G.
, ‘Death and life in a colonial immigrant city: a demographic analysis of Philadelphia’, Journal of Economic History, 37 (1977), pp. 863–89.1163225010.1017/s0022050700094729

Smith, R. M.
, ‘Population and its geography in England 1500–1730’, in DodgshonR. A. and ButlinR. A., eds., An historical geography of England and Wales (1978), pp. 199–238.

Söderberg, J.
, 
Jonsson, U.
, and 
Persson, C.
, A stagnating metropolis: the economy and demography of Stockholm, 1750–1850 (Cambridge, 1991).

Sussman, G. D.
, ‘Parisian infants and Norman wet nurses in the early nineteenth century: a statistical study’, Journal of Interdisciplinary History, 7 (1977), pp. 637–53.11632284

Swedlund, A. C.
 and 
Donta, A. K.
, ‘Scarlet fever epidemics in the nineteenth century: a case of evolved pathogenic virulence?’, in HerringD. A. and SwedlundA. C., eds., Human biologists in the archives (Cambridge, 2003), pp. 159–77.

Sykes, W. H.
, 
Sandon, Lord
, 
Porter, G. R.
, 
Heywood, J.
, 
Alison, W. P.
, and 
Chadwick, E.
, ‘Report on the vital statistics of large towns in Scotland’, 12th annual meeting of the British Association for the Advancement of Science (1842), pp. 121–204.

Szreter, S.
, ‘Economic growth, disruption, deprivation, disease, and death: on the importance of the politics of public health for development’, Population and Development Review, 23 (1997), pp. 693–728.

Szreter, S.
, ‘The population health approach in historical perspective’, American Journal of Public Health, 93 (2003), pp. 421–31.1260448610.2105/ajph.93.3.421PMC1449802

Szreter, S.
 and 
Mooney, G.
, ‘Urbanisation, mortality, and the standard of living debate: new estimates of the expectation of life at birth in nineteenth‐century British cities’, Economic History Review, LI (1998), pp. 84–112.

Torres, C.
, 
Canudas‐Romo, V.
, and 
Oeppen, J.
, ‘The contribution of urbanization to changes in life expectancy in Scotland, 1861–1910’, Population Studies, 73 (2019), pp. 387–404.3070202610.1080/00324728.2018.1549746

Vinovskis, M. A.
, ‘Mortality rates and trends in Massachusetts before 1860’, Journal of Economic History, 32 (1972), pp. 184–213.1163225310.1017/s002205070007546x

Vögele, J.
, Urban mortality change in England and Germany, 1870–1913 (Liverpool, 1998).

de Vries, J.
, European urbanization, 1500–1800 (Cambridge, Mass., 1984).

de Vries, J.
, ‘Problems in the measurement, description, and analysis of historical urbanization’, in van der WoudeA., HayamiA., and de VriesJ., eds., Urbanization in history: a process of dynamic interactions (Oxford, 1990), pp. 43–60.

Watt, A.
, The Glasgow bills of mortality for 1841 and 1842 (Glasgow, 1844).

Weir, D. R.
, ‘Economic welfare and physical well‐being in France, 1750–1990’, in SteckelR. H. and FloudR., eds., Health and welfare during industrialization (1997), pp. 161–200.

Wheatcroft, S.
, ‘Eastern Europe (Russia and the USSR)’, in AlfaniG. and Ó’GrádaC., eds., Famine in European history (Cambridge, 2017), pp. 212–39.

Woods, R.
, ‘The effect of population redistribution on the level of mortality in nineteenth‐century England and Wales’, Journal of Economic History, 45 (1985), pp. 645–51.1161731110.1017/s0022050700034549

Woods, R.
, ‘On the historical relationship between infant and adult mortality’, Population Studies, 47 (1993), pp. 195–219.1161305510.1080/0032472031000146976

Woods, R.
, ‘Causes of death in England and Wales, 1851–1860 to 1891–1900: the decennial supplements’ [data collection] (1997), UK Data Service, SN 3552.

Woods, R.
, The demography of Victorian England and Wales (Cambridge, 2000).

Woods, R.
 and 
Shelton, N.
, An atlas of Victorian mortality (Liverpool, 1997).

van der Woude, A. M.
, ‘Population developments in the Northern Netherlands (1500–1800) and the validity of the “urban graveyard” effect’, Annales de Demographie Historique (1982), pp. 55–75.11629029

Wrigley, E. A.
, ‘A simple model of London's importance in changing English society and economy 1650–1750’, Past and Present, 37 (1967), pp. 44–70.

Wrigley, E. A.
, 
Davies, R. S.
, 
Oeppen, J. E.
, and 
Schofield, R. S.
, English population history from family reconstitution 1580–1837 (Cambridge, 1997).

Wrigley
E. A.
 and 
Schofield
R. S.
, ‘English population history from family reconstitution: summary results 1600–1799’, Population Studies, 37 (1983), pp. 157–84.10.1080/00324728.1983.1040874511631033

Wrigley
E. A.
 and 
Schofield
R. S.
, The population history of England 1541–1871 (2nd edn., Cambridge, 1989).10.1080/0307102830856755811624725Official publication1
*8th–23rd Annual Reports of the Registrar‐General*
(P.P. 1847/8, XXV; 1849, XXI; 1850, XX; 1851, XXII; 1852, XVIII; 1852/3, XL; 1854, XIX; 1854/5, XV; 1856, XVIII; 1857, XXII; 1857/8, XXIII; 1859, XII; 1860, XXIX; 1861, XVIII; 1862, XVII).
